# The impact of baseline glomerular filtration rate on subsequent changes of glomerular filtration rate in patients with chronic kidney disease

**DOI:** 10.1038/s41598-021-86955-z

**Published:** 2021-04-12

**Authors:** Yi-Chih Lin, Tai-Shuan Lai, Shuei-Liong Lin, Yung-Ming Chen, Tzong-Shinn Chu, Yu-Kang Tu

**Affiliations:** 1 Institute of Epidemiology and Preventive Medicine, College of Public Health, National Taiwan University, Room 501, No. 17, Xu-Zhou Road, Taipei, 100 Taiwan; 2 Division of Nephrology, Department of Internal Medicine, National Taiwan University Hospital and College of Medicine, No. 7, Chung-Shan South Road, Taipei, 100 Taiwan; 3 Department of Medicine, National Taiwan University Hospital Jinshan Branch, New Taipei City, Taiwan; 4 Graduate Institute of Physiology, National Taiwan University College of Medicine, Taipei, Taiwan; 5 Department of Integrated Diagnostics & Therapeutics, National Taiwan University Hospital, Taipei, Taiwan; 6 Research Center for Developmental Biology and Regenerative Medicine, National Taiwan University, Taipei, Taiwan; 7 Department of Dentistry, National Taiwan University Hospital, Taipei, Taiwan; 8 Research Center of Big Data and Meta-Analysis, Wan Fang Hospital, Taipei Medical University, Taipei, Taiwan

**Keywords:** Chronic kidney disease, Statistics

## Abstract

Higher baseline glomerular filtration rate (GFR) may yield subsequent steeper GFR decline, especially in patients with diabetes mellitus (DM). However, this correlation in patients with chronic kidney disease (CKD) and the presence or absence of DM remains controversial. We conducted a longitudinal cohort study in a single medical center between 2011 and 2018. Participants with CKD stage 1 to 3A were enrolled and divided into DM groups and non-DM groups, and then followed up at least every 6 months. We used a linear mixed regression model with centering time variable to overcome the problem of mathematical coupling in the analysis of the relation between baseline GFR and the changes, and compared the results from correct and incorrect specifications of the mixed models. A total number of 1002 patients with 285 diabetic and 717 non-diabetic persons was identified. The linear mixed regression model revealed a significantly negative correlation between baseline GFR and subsequent GFR change rate in both diabetic group and non-diabetic group (r =  − 0.44 [95% confidence interval [CI], − 0.69 to − 0.09]), but no statistical significance in non-diabetic group after within-subject mean centering of time variable (r =  − 0.09 [95% CI, − 0.41 to 0.25]). Our study showed that higher baseline GFR was associated with a subsequent steeper GFR decline in the DM group but not in the non-DM group among patients with early-stage CKD. Exact model specifications should be described in detail to prevent from a spurious conclusion.

## Introduction

Chronic kidney disease (CKD), affecting more than 10% of the people worldwide, becomes a growing public health issue^1–3^. With the progression of CKD, the risk of developing end-stage renal disease (ESRD) increases gradually and so do the cardiovascular complications and deaths^4,5^. Traditional risk factors of CKD progression include hypertension, diabetes mellitus (DM), and higher body mass index (BMI)^6^; however, these factors do not provide a very accurate prediction of the speed of glomerular filtration rate (GFR) decline. Some patients’ GFR show rapid decline, while others remain stable^7,8^.

Several investigations indicated that higher baseline GFR, such as renal hyperfiltration, may relate to subsequent rapid GFR decline. Higher baseline GFR have been found in early stages of DM if hyperglycemia was not well controlled^9–13^. The pathophysiology of hyperfiltration in DM comprised several possible mechanisms, such as ultrastructural changes caused by imbalanced release of cytokines and growth factors, and disturbance of vascular tone due to an imbalance of vasoactive humoral factors in response to hyperglycemia^12,14,15^. Moreover, most observational studies and meta-analyses have shown a significant relation between higher baseline GFR and subsequent rapid GFR decline in patients with DM^16–19^.

The phenomenon of higher baseline GFR was also found in people with intolerant fasting glucose, obesity, pregnancy, or high protein diet^20–22^. However, there is limited information about the relation between higher baseline GFR and subsequent GFR decline in patients without DM. A recent study showed that higher baseline GFR was significantly related to more rapid decline in GFR over time in patients without DM by using a linear mixed regression model with a random intercept and slope, i.e. the variations in the baseline GFR and in the changes in GFR respectively, to resolve the statistical issue of mathematical coupling^17^. Mathematical coupling, defined as one variable containing the whole or part of another, could yield a spurious correlations between two variables irrespective of any true association, thereby leading to questionable conclusions^3,17,23–25^. Although linear mixed model can tackle the issue of mathematical coupling, correct specification of the random effects is essential for yielding a meaningful interpretation of the correlation between the random intercept and slope^26^. However, previous studies did not always describe their model specifications in detail, and it is, therefore, unclear whether the issue of mathematical coupling has indeed been resolved.

To investigate the relationship between the baseline GFR and subsequent GFR change, we used an early stages of CKD cohort comprising patients with DM and without DM. We used linear mixed model to overcome the mathematical coupling in the analysis of the relation between the baseline GFR and its changes. We also compared results from correct and incorrect specifications of mixed models to assess the impact of mathematical coupling. We hypothesized that higher baseline GFR is a risk factor of a rapid GFR decline in patients with DM but not in those without DM.

## Methods and materials

### Study population

This study is a retrospective analysis of data from a single medical center, National Taiwan University Hospital. Patients, who agreed to participate the early CKD program between 2011 and 2018, were included, if their ages were between 18 and 80 years and were diagnosed with chronic kidney disease stage 1 to 3A, which was defined as a 45 ml/min/1.73 m^2^ < GFR < 60 ml/min/1.73 m^2^ or GFR ≥ 60 ml/min/1.73 m^2^ with a urinary protein-to-creatinine ratio (UPCR) ≥ 150 mg/g or significant findings of renal pathology for at least 3 months^27,28^. Patients, who had acute kidney injury within three months, Child Pugh class B to C liver cirrhosis, or terminal malignancies, were excluded. We then divided the cohort into DM (including type 1 and type 2 DM) and non-DM groups to proceed with statistical analyses. This study has been approved by the Research Ethics Committee of the National Taiwan University Hospital (202006020RINB). As this study was retrospective and observational, participants written informed consent was waived by the Research Ethics Committee of the National Taiwan University Hospital.

### Early chronic kidney disease program

The early CKD program was initiated by the National Health Insurance Bureau, the Ministry of Health and Welfare in Taiwan, to care patients with CKD stages 1 to 3a in high-risk population since 2011^29^. The high-risk population of CKD progression includes people of being older than 65 years old, family history of CKD, diabetes mellitus, and hypertension.

These patients were invited to join this program on their visits to outpatient clinics. A comprehensive educational program, consisting of basic knowledge of chronic kidney disease, risk factors, lifestyle modification and medical treatment, was provided. The participants returned to the clinic every 3–6 months, according to the clinical presentations of patients and judgments of primary care physicians. They attended classes of the educational programs and undertook routine laboratory tests at least every 6 months. Once patients’ GFR was lower than 45 ml/min/1.73 m^2^ or UPCR≧1000 mg/gm, they were transferred to the Pre-ESRD program for further management^29^.

### Data collection

Basic personal information of age, sex and underlying comorbidity were recorded. Body height, weight, blood pressure and biochemical data, including serum creatinine, low-density lipoprotein cholesterol, fasting glucose and HbA1c (only for DM patients) and UPCR were recorded at baseline and every 3 to 6 months. The estimated GFR (eGFR) was calculated by the Taiwanese MDRD equation [1.309 × (186 × (serum creatinine)^−1.154^ × Age ^−0.203^ × 0.742 (if female)) ^0.912^] , which was developed by using linear regression of the difference on the average of log-transformed inulin clearance and the MDRD, CKD-Epidemiology Collaboration (CKD-EPI) equations, and has been validated and shown to be more accurate and precise than MDRD-4 variables and CKD-EPI equations for Taiwanese adults^28,30^.

### Statistical analyses

We used the mean ± standard deviation (SD) to summarize continuous variables, and relative frequency for categorical variables. The differences between the two groups were analyzed by using the t-test or the chi-squared test. Variables with a non-normal distribution were analyzed by the Mann–Whitney U test. A two-level linear mixed regression model with the random intercept and slope was used to analyze eGFR measurements and solving the statistical problems of mathematical coupling. The dependent variable was the absolute value of eGFR. The time variable was the duration of observation starting from the date of the first visit up to that of the last visit in nephrology outpatient department. To test the appropriate null hypothesis in multivariable linear mixed regression model, we undertook within-subject mean centering for the duration of observation to correct for the effects of mathematical coupling between random intercept and slope^23–26^. The basic linear mixed model is written as:

$$eGF{R}_{ij}={b}_{0j}+{b}_{1j}Tim{e}_{ij}+{e}_{ij}$$

$${b}_{0j}={\beta }_{0}+{u}_{0j}$$

$${b}_{1j}={\beta }_{1}+{u}_{1j}$$

$$\left(\genfrac{}{}{0pt}{}{{u}_{0j}}{{u}_{1j}}\right)\sim N\left(\begin{array}{cc}0,&{\varvec{\Sigma}}\end{array}\right),{\varvec{\Sigma}}=\left(\begin{array}{cc}{\sigma }_{u0}^{2}& {\sigma }_{u01}\\ {\sigma }_{u01}& {\sigma }_{u1}^{2}\end{array}\right)$$

$${e}_{ij}\sim N\left(\begin{array}{cc}0,& {\sigma }_{e}^{2}\end{array}\right)$$

where $eGF{R}_{ij}$ is the observed eGFR for the *j*th patient on *i*th occasion; ${b}_{0j}$ is the intercept for the *j*th patient ${\beta }_{0}$ is the average intercept for the whole patient population, and ${u}_{0j}$ is the random intercept, i.e. the variations in the intercepts of the whole patient population, which is assumed to follow a normal distribution with the mean of zero and variance of ${\sigma }_{u0}^{2}$; *Time* is the centered time variable, i.e. the centered duration of observation in years for each patient; ${b}_{1j}$ is the slope for the *j*th patient ${\beta }_{1}$ is the average slope for the whole patient population, and ${u}_{1j}$ is the random slope, i.e. the variations in the slopes of the whole patient population, which is assumed to follow a normal distribution with the mean of zero and variance of ${\sigma }_{u1}^{2}$; and ${e}_{ij}$ is the residual error term for $eGF{R}_{ij}$. ${u}_{0j}$ and ${u}_{1j}$ follow a bivariate normal distribution, and the parameter ${\sigma }_{u01}$ is the covariance between random intercept and slope and can be used to calculate the correlation *r* between the baseline eGFR and the subsequent changes in eGFR by using the formulae: $r=\frac{{\sigma }_{u01}}{\sqrt{{\sigma }_{u0}^{2}*{\sigma }_{u1}^{2}}}$. The effect of an independent variable on GFR change rate in mL/min per year was evaluated by using 2-way interaction between the independent variables and the centered time variable. The relation between baseline eGFR (ml/min/1.73 m^2^) and succeeding eGFR changes (ml/min/1.73 m^2^ per year) was evaluated by the correlation between the random intercept and slopes. We also conducted the same analyses without centering the time variables to assess the impact of mathematical coupling on the estimation of the correlation between the baseline eGFR and the change. We performed covariates adjustment, including sex and age in model 1, then plus BMI and systolic blood pressure (SBP) in model 2, and added current smoking status, HbA1c (only in diabetes mellitus group) and urine protein-creatinine ratio in model 3. A *P*-value < 0.05 was considered statistically significance. The statistical software Stata version 14 (Stata Corp College Station, Texas, USA) (https://www.stata.com/) was used for data analysis. All methods carried out were in accordance with relevant guidelines and national legal regulations.

## Results

### Baseline characteristics of study population

**Figure 1:**
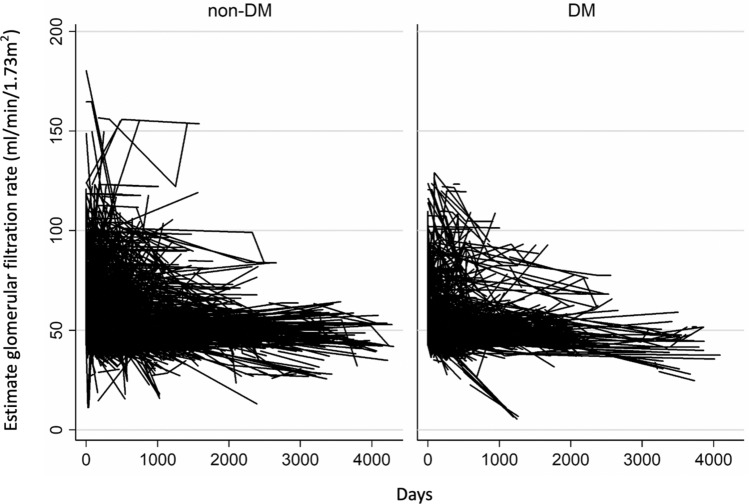
The graph of estimated glomerular filtration rate (eGFR) change without within-subject mean centering for the time variable.

The baseline characteristics of diabetes mellitus and non-diabetes mellitus groups is shown in Table 1. A total number of 1002 patients with early-stage CKD (717 in non-DM group and 285 in DM group, respectively) and 7621 nephrology clinic visits were identified between January 1, 2011, and December 31, 2018. More than 70 percent of patients in both DM and non-DM groups were male (79.6% and 72.9%, respectively). Patients in DM group were 4.4 years older than those in non-DM group (68.0 vs 63.6 years old, p < 0.001) and had a higher BMI (mean BMI 26.0 vs 24.8 kg/m^2^, p < 0.001, respectively). Mean baseline eGFR was 58.5 ± 18.1 (SD) ml/min/1.73 m^2^ in the non-DM group and 57.4 ± 16.3 (SD) ml/min/1.73 m^2^ in DM group by the Taiwanese MDRD equation. The eGFR declined gradually in both DM and non-DM groups (Fig. 1), but the lengths of follow-up varied greatly among participants.

**Table 1:** Baseline characteristics of study population.

	Non-DM group (N = 717)	DM group (N = 285)	P value
Age (year)	63.6 ± 15.5	68.0 ± 11.0	0.0004
Sex (male)	523 (72.9%)	227 (79.6%)	0.0335
Height (cm)	164.1 ± 7.81	163.9 ± 7.9	0.8233
Weight (kg)	66.9 ± 11.8	70.1 ± 12.6	0.0001
BMI (kg/m^2^)	24.8 ± 3.7	26.0 ± 4.0	< 0.0001
Current smoker (yes)	78 (10.9%)	62 (21.8%)	< 0.0001
Alcohol drink (yes)	49 (6.8%)	46 (16.1%)	< 0.0001
SBP (mmHg)	128.8 ± 14.4	130.4 ± 15.9	0.100
DBP (mmHg)	76.7 ± 9.5	75.5 ± 9.4	0.046
Serum creatinine (mg/dL)	1.2 ± 0.31	1.2 ± 0.28	0.861
LDL cholesterol (mg/dL)	105.4 ± 32.0	96.2 ± 27.4	< 0.0001
Glucose (AC)(mg/dL)	110.2 ± 45.1	135.5 ± 46.7	< 0.0001
HbA1c (%)	6.3 ± 1.3	7.1 ± 1.4	< 0.0001
Urine protein-creatinine ratio (10 mg/day)	39.4 ± 83.2	39.9 ± 42.6	0.030
Baseline eGFR (ml/min/1.73m^2^)	58.5 ± 18.1	57.4 ± 16.3	0.434

Estimates are given as number (percent) and mean ± standard deviation.

*DM* diabetes mellitus, *BMI* body-mass index, *SBP* systolic blood pressure, *DBP* diastolic blood pressure, *LDL* low-density lipoprotein, Hemoglobin A1c, *UPCR* urine protein-creatinine ratio, *eGFR* estimated glomerular filtration rate.

### Fixed effects of risk factors on the baseline eGFR and changes of eGFR

Our multivariable mixed model (model 3) showed that males and older age had a significantly negative association with baseline eGFR; the average baseline eGFR of men was 4.61 ml/min/1.73 m^2^ lower than that of women, and the average baseline eGFR decreased by 0.50 ml/min/1.73 m^2^ when a patient’s age increased by 1 year. UPCR had a significantly negative relationship to the changes of eGFR in the DM group; the average change in eGFR decreased by 0.10 ml/min/1.73 m^2^ as UPCR increased by 10 mg/g (Table 2). In the non-DM group, male and older age also had a negative association with baseline eGFR; the average baseline eGFR of men was 10.85 ml/min/1.73 m^2^ lower than that of women, and the average baseline eGFR decreased by 0.44 ml/min/1.73 m^2^ when a patient’s age increased by 1 year, and higher systolic blood pressure showed greater changes in eGFR; the average change in eGFR increased by 0.02 ml/min/1.73 m^2^ when systolic blood pressure increased by 1 mmHg (Table 3).

**Table 2:** Fixed effects of risk factors on baseline eGFR and changes of eGFR in patients with DM.

Fixed effects	Model 1		Model 2		Model 3	
	Coefficient, ml/min/1.73m^2^ (95% CI)	*P *value	Coefficient, ml/min/1.73m^2^ (95% CI)	*P *value	Coefficient, ml/min/1.73m^2^ (95% CI)	*P *value
**Effects on baseline eGFR (intercept)**						
Sex (male)	− 5.47* (− 9.51 to − 1.43)	0.008	− 6.18* (− 10.27 to − 2.08)	0.003	− 4.61* (− 9.04 to − 0.18)	0.04
Age (per 1 year older)	− 0.55* (− 0.69 to − 0.40)	< 0.001	− 0.51* (− 0.66 to − 0.37)	< 0.001	− 0.50* (− 0.66 to − 0.35)	< 0.001
BMI (per kg/m^2^)			0.11 (− 0.11 to 0.33)	0.32	0.08 (− 0.19 to 0.34)	0.58
SBP (per 1 mmHg greater)			0.02 (− 0.01 to 0.05)	0.13	0.01 (− 0.03 to 0.05)	0.57
Smoking (yes vs no)					1.74 (− 1.85 to 5.33)	0.34
HbA1c (per 1%)					− 0.25 (− 0.93 to 0.43)	0.47
UPCR (per 10 mg/g)					0.01 (-0.01 to 0.02)	0.09
**Effects on changes of eGFR (slope)**						
Sex (male)	− 0.17 (− 1.46 to 1.12)	0.80	− 0.20 (− 1.65 to 1.26)	0.79	0.66 (− 1.20 to 2.52)	0.49
Age (per 1 year older)	0.01 (− 0.03 to 0.05)	0.60	0.03 (− 0.02 to 0.07)	0.22	0.01 (− 0.04 to 0.05)	0.86
BMI (per kg/m^2^)			− 0.08 (− 0.17 to 0.01)	0.05	− 0.06 (− 0.16 to 0.04)	0.25
SBP (per 1 mmHg greater)			− 0.01 (− 0.02 to 0.01)	0.74	0.01 (− 0.02 to 0.03)	0.83
Smoking (yes vs no)					− 0.53 (− 1.58 to 0.52)	0.33
HbA1c (per 1%)					− 0.06 (− 0.45 to 0.33)	0.78
UPCR (per 10 mg/g greater)					− 0.01* (− 0.02 to − 0.01)	< 0.001

Model 1: sex and baseline age. Model 2: sex, baseline age, BMI, and SBP. Model 3: sex, baseline age, BMI, SBP, smoking, HbA1c, and UPCR.

*eGFR* estimated glomerular filtration rate, *DM* diabetes mellitus, *CI* confidence interval, *BMI* body mass index, *SBP* systolic blood pressure, *HbA1c* hemoglobin A1c, *UPCR* urine protein-creatinine ratio.

*P < 0.05.

**Table 3:** Fixed effects of risk factors with baseline eGFR and changes of eGFR in patients without DM.

Fixed effects	Model 1		Model 2		Model 3	
	Coefficient, ml/min/1.73m^2^ (95% CI)	*P value*	Coefficient, ml/min/1.73m^2^ (95% CI)	*P value*	Coefficient, ml/min/1.73m^2^ (95% CI)	*P value*
**Effects on baseline eGFR (intercept)**						
Sex (male)	− 11.26* (− 13.98 to − 8.54)	< 0.001	− 11.14* (− 13.51 to − 8.77)	< 0.001	-10.85* (− 13.26 to − 8.44)	< 0.001
Age (per 1 year older)	− 0.54* (− 0.62 to − 0.45)	< 0.001	− 0.43* (− 0.49 to − 0.36)	< 0.001	− 0.44* (− 0.51 to − 0.37)	< 0.001
BMI (per kg/m^2^)			− 0.16 (− 0.36 to 0.04)	0.12	− 0.18 (− 0.38 to 0.03)	0.09
SBP (per 1 mmHg greater)			0.01 (− 0.01 to 0.04)	0.30	0.002 (− 0.02 to 0.03)	0.90
Smoking (yes vs no)					0.43 (− 2.65 to 3.52)	0.78
UPCR (per 10 mg/g)					− 0.002 (− 0.01 to 0.002)	0.28
**Effects on changes of eGFR (slope)**						
Sex (male)	1.41* (0.77 to 2.06)	< 0.001	0.80* (0.10 to 1.50)	0.03	0.59 (− 0.02 to 1.20)	0.06
Age (per 1 year older)	− 0.01 (− 0.02 to 0.02)	0.71	− 0.01 (− 0.02 to 0.02)	0.91	0.01 (− 0.01 to 0.02)	0.67
BMI (per kg/m^2^)			0.06 (− 0.02 to 0.13)	0.14	0.03 (− 0.04 to 0.10)	0.39
SBP (per 1 mmHg greater)			0.02* (0.01 to 0.03)	0.02	0.02* (0.01 to 0.04)	0.01
Smoking (yes vs no)					− 0.25 (− 0.97 to 0.46)	0.49
UPCR (per 10 mg/g greater)					0.001 (− 0.01 to 0.01)	0.77

Model 1: sex and baseline age. Model 2: sex, baseline age, BMI, and SBP. Model 3: sex, baseline age, BMI, SBP, smoking, HbA1c, and UPCR.

*eGFR* estimated glomerular filtration rate, *DM* diabetes mellitus, *CI* confidence interval, *BMI* body mass index, *SBP* systolic blood pressure, *UPCR* urine protein-creatinine ratio.

*P < 0.05.

### Association between baseline eGFR and subsequent eGFR change

**Figure 2:**
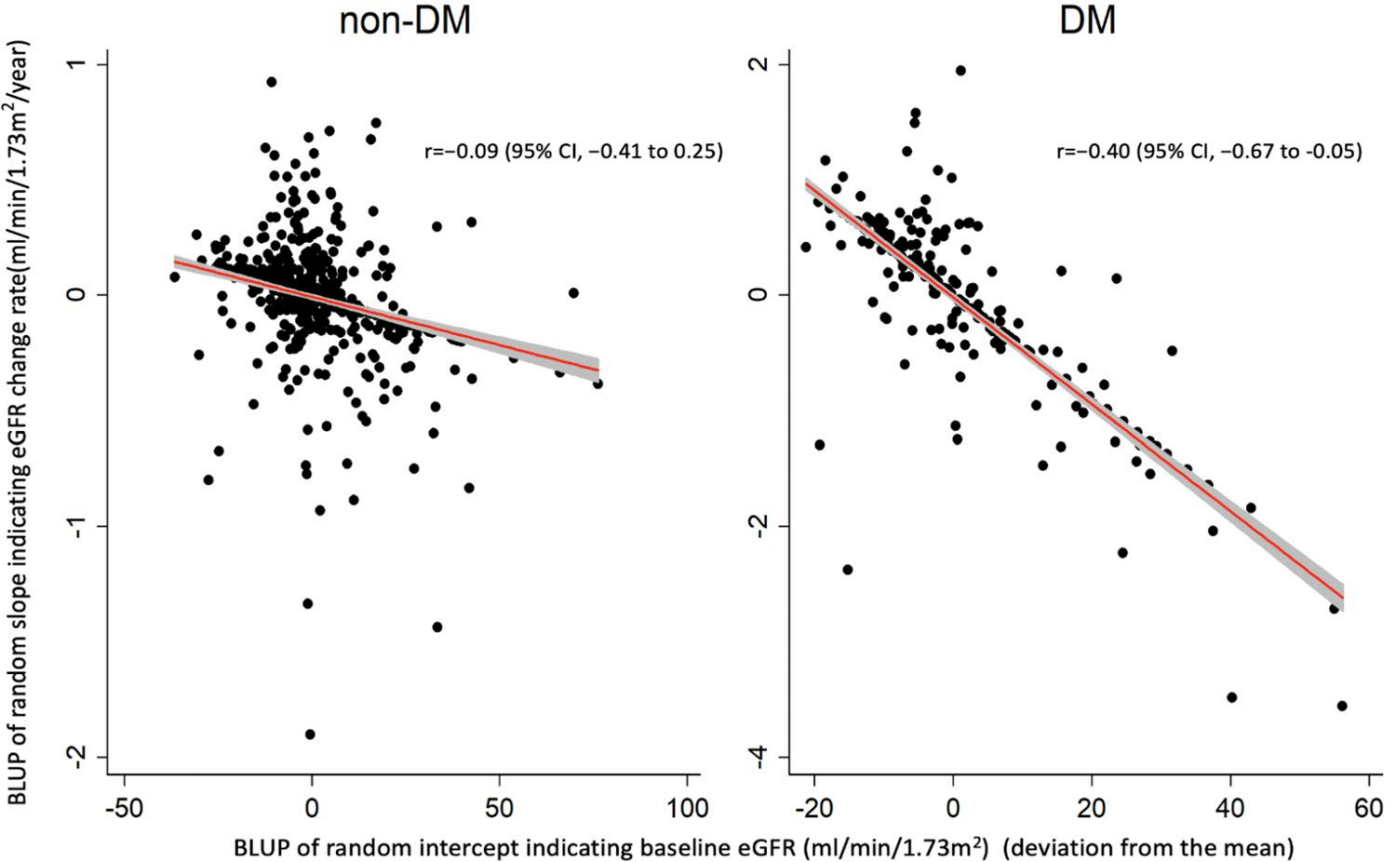
The relation between baseline glomerular filtration rate (GFR) and GFR change rate. The figure shows the best linear unbiased predictors (BLUPs) of random slopes and intercepts, indicating the relation between baseline GFR (the random intercept) and GFR change rate (random slope) in a linear mixed model after using within-subject mean centering for the time variable. DM, diabetes mellitus; CI, confidence interval. Stata version 14 (Stata Corp College Station, Texas, USA) (https://www.stata.com/).

In the DM group, a significantly consistent negative correlation between GFR at baseline (random intercept) and GFR change rate (random slope) was found after multivariable adjustment (r =  − 0.46 [95% CI, − 0.62 to − 0.27] in model 1, r =  − 0.51 [95% CI, − 0.68 to − 0.30] in model 2, and r =  − 0.65 [95% CI, − 0.81 to − 0.39] in model 3, respectively). After within-subject mean centering for the time variable, the correlations decreased substantially (r =  − 0.25 [95% CI, − 0.44 to − 0.04] in model 1, r =  − 0.30 [95% CI, − 0.51 to − 0.05] in model 2, and r =  − 0.40 [95% CI, − 0.67 to − 0.05] in model 3, respectively) (Table 4) (Fig. 2).

**Table 4:** Correlation between baseline eGFR and eGFR decreased rates (random effects) in patients with DM.

Random effects	Model 1	Model 2	Model 3
	Estimate (95% CI)	Estimate (95% CI)	Estimate (95% CI)
**Without within-subject mean centering for the time variable**			
SD of slope, mL/min/y (GFR change)	2.27 (1.80 to 2.86)	2.08 (1.61 to 2.68)	1.48 (1.06 to 2.07)
SD of intercept, mL/min	17.76 (16.29 to 19.36)	17.70 (16.20 to 19.34)	14.34 (13.01 to 15.81)
SD of residuals	6.43 (6.22 to 6.65)	6.11 (5.80 to 6.42)	5.07 (4.70 to 5.47)
Correlation (intercept, slope)	− 0.46* (− 0.62 to − 0.27)	− 0.51* (− 0.68 to − 0.30)	− 0.65* (− 0.81 to − 0.39)
**Using within-subject mean centering for the time variable**			
SD of slope, mL/min/y (GFR change)	1.99 (1.62 to 2.46)	1.94 (1.53 to 2.46)	1.50 (1.03 to 2.17)
SD of intercept, mL/min	13.86 (12.73 to 15.09)	13.74 (12.59 to 15.00)	13.54 (12.30 to 14.91)
SD of residuals	5.28 (5.11 to 5.45)	4.93 (4.69 to 5.19)	5.12 (4.74 to 5.52)
Correlation (intercept, slope)	− 0.25* (− 0.44 to − 0.04)	− 0.30* (− 0.51 to − 0.05)	− 0.40* (− 0.67 to − 0.05)

Model 1: sex and baseline age. Model 2: sex, baseline age, BMI, and SBP. Model 3: sex, baseline age, BMI, SBP, smoking, HbA1c, and UPCR.

*eGFR* estimated glomerular filtration rate, *DM* diabetes mellitus, *CI* confidence interval, *BMI* body mass index, *SBP* systolic blood pressure, *HbA1c* hemoglobin A1c, *UPCR* urine protein-creatinine ratio.

*P < 0.05.

In the non-DM group, multivariable mixed linear regression model also showed a significantly negative correlation between GFR at baseline and GFR change rate (r =  − 0.22 [95% CI, − 0.36 to − 0.06] in model 1, r =  − 0.36 [95% CI, − 0.52 to − 0.17] in model 2, and r =  − 0.44 [95% CI, − 0.69 to − 0.09] in model 3, respectively). However, after within-subject mean centering for the time variable, the negative correlation between GFR at baseline and GFR change became much smaller and no longer statistically significant (r = 0.05 [95% CI, − 0.11 to 0.21] in model 1, r =  − 0.06 [95% CI, − 0.26 to 0.15] in model 2, and r =  − 0.09 [95% CI, − 0.41 to 0.25] in model 3, respectively) (Table 5) (Fig. 2).

**Table 5:** Correlation between baseline eGFR and eGFR decreased rates (random effects) in patients without DM.

Random effects	Model 1	Model 2	Model 3
	Estimate (95% CI)	Estimate (95% CI)	Estimate (95% CI)
**Without within-subject mean centering for the time variable**			
SD of slope, mL/min/y (GFR change)	2.37 (2.04 to 2.74)	1.83 (1.42 to 2.36)	0.73 (0.43 to 1.23)
SD of intercept, mL/min	17.74 (16.78 to 18.75)	17.56 (16.58 to 18.61)	14.25 (13.43 to 15.11)
SD of residuals	7.52 (7.36 to 7.68)	7.25 (7.01 to 7.49)	5.97 (5.76 to 6.18)
Correlation (intercept, slope)	− 0.22* (− 0.36 to − 0.06)	− 0.36* (− 0.52 to − 0.17)	− 0.44* (− 0.69 to − 0.09)
**Using within-subject mean centering for the time variable**			
SD of slope, mL/min/y (GFR change)	2.09 (1.80 to 2.41)	1.64 (1.27 to 2.11)	0.83 (0.49 to 1.39)
SD of intercept, mL/min	14.16 (13.41 to 14.95)	13.90 (13.13 to 14.71)	14.04 (13.25 to 14.87)
SD of residuals	6.15 (6.02 to 6.28)	5.84 (5.65 to 6.05)	5.94 (5.73 to 6.16)
Correlation (intercept, slope)	0.05 (− 0.11 to 0.21)	− 0.06 (− 0.26 to 0.15)	− 0.09 (− 0.41 to 0.25)

Model 1: sex and baseline age. Model 2: sex, baseline age, BMI, and SBP. Model 3: sex, baseline age, BMI, SBP, smoking, HbA1c, and UPCR.

*eGFR* estimated glomerular filtration rate, *DM* diabetes mellitus, *CI* confidence interval, *BMI* body mass index, *SBP* systolic blood pressure, *UPCR* urine protein-creatinine ratio.

*P < 0.05.

## Discussion

To our best knowledge, this study is the first to explore the relation between baseline eGFR and subsequent GFR decline in DM and non-DM patients with early-stage CKD in a long-term population-based cohort. After multivariable adjustment, we found that male and increased age had a significantly negative effect on the baseline eGFR, and only UPCR showed a significant relation to the decrease in eGFR in patients with CKD and DM. In patients with CKD and non-DM, males and older age also showed a significantly negative association with the baseline eGFR but males (in model 1 and 2) and higher systolic pressure were associated with a significantly smaller decrease in eGFR. Naïve analyses without centering of the time variable found a significantly negative correlation between GFR at baseline and GFR change in DM and non-DM patients with CKD. After within-subject mean centering, a significant but moderate correlation were observed only in DM patients with CKD. This indicates the impact of mathematical coupling on the estimation of the correlation between GFR at baseline and GFR change cannot be overlooked, and this is consistent with findings in the previous methodological studies^23,25,31–33^.

It is still controversial whether hyperfiltration with higher baseline GFR is related to subsequent GFR decline. Some studies revealed that hyperfiltration with higher eGFR was associated with more rapid eGFR decline in patients with type 1 or type 2 DM^16–19,34–36^. Moreover, Melsom et al. showed that this significant correlation existed not only in patients with DM but also in those without DM^17^. In contrast, no substantial correlation between baseline GFR and subsequent GFR decline was also found in other investigations^37,38^. This inconsistency may be attributed to considerable GFR variations over time, such as age-related GFR decline, GFR measurements, and inappropriate statistical methods for assessing the relation between GFR at baseline and its changes^39,40^. In our study, a negative relation between the baseline eGFR and subsequent decline was found only in patients with DM but not in patients without DM.

The accurate measurement of GFR should examine the clearance of materials which are only through renal filtration, such as iohexol, iothalamate, inulin, etc.^41^. However, the estimated GFR was more readily available and cost-effective in the clinical practice than GFR via direct measurement, which was difficult to replicate due to different physiological conditions and great variations over time. Two of the most common equations for estimating GFR were MDRD-4 variables and CKD-EPI worldwide. However, the MDRD-4 equation was created by using data from Caucasians and African Americans with CKD and is likely to underestimate GFR, when eGFR greater than 60 mL/min/ 1.73 m^2^^42,43^. The KDIGO 2012 guidelines recommend using the CKD-EPI equation in adults, unless an alternative equation has been shown to be more accurate in the specific population^44^. In our study, we used a corrected MDRD equation (Taiwanese MDRD), because it has been shown to have better precision and a lower bias than the equations of the MDRD-4 and CKD-EPI in Taiwanese population^30^.

Analysis of the relation between the baseline and changes suffered from mathematical coupling, which gives rise to misleading results and invalid testing of the null hypothesis^23,25^. Some studies applied a linear mixed regression model with random intercept and slope to resolve this statistical issue and to attain correct results^17^. However, the correct null hypothesis for testing the correlation between the baseline value and subsequent change is not zero, because these two variables have an underlying mathematical relation by sharing a common component of the baseline value^23,25^. It has been shown that centering the time variable could overcome this underlying mathematical relation and then attain an accurate result of null hypothesis test^23–25,45^. In this study, we undertook within-subject mean centering for the time variable to investigate the relationship between the baseline eGFR and its changes. We observed that the negative correlations are always attenuated after centering when compared with those given by the conventional approach without centering, in DM and non-DM patients with CKD. Moreover, in non-DM patients, this negative correlations were no longer statistically significant, after correct methodologies were used. This implied that higher baseline eGFR or renal hyperfiltration may be only a subclinical indicator but not the major cause of renal damage in patients with DM and early-stage CKD. Furthermore, when patients’ GFR was lower than 45 ml/min/1.73 m^2^, they would be transferred to the Pre-ESRD program, and their eGFR values were no longer being included in our analysis. This may lead to the truncation of low eGFR values, resulting in the reduction in the variances of follow-up eGFR and the over-estimation of the negative relation between the baseline eGFR and its changes. Our study suggests that previous evidence on the relation between the baseline eGFR and the decline in eGFR should be interpreted with great cautions and may require reevaluation.

### Limitations

Our study has some limitations. First, we included a relatively small number of participants, especially in patients with DM. Despite this problem, our study collected sufficient data of repeated measurements during the long-term follow-up to achieve robust inference. Second, our study included a higher proportion of men and elderly in DM and non-DM groups. According to previous national study, near two thirds of adults with CKD was more than 60 years old (63.3%), and men had a higher prevalence of early-stage CKD than did women (11.7% versus 9.9%)^46^. These data showed a similar distribution to our study. Therefore, it needs to be cautious to apply findings in our study to other populations with different sex proportions or age distributions. Third, the mean eGFR in our study was lower than the traditional definition of hyperfiltration with high GFR. The focus of our study is not the consequence of renal hyperfiltration but proper analyses of the relation between higher baseline GFR and subsequent GFR change. Moreover, the number of nephrons varied among individuals and usually decreased with age or renal injury^39^. Glomerular hyperfiltration or single-nephron hyperfiltration in people with fewer numbers of nephrons may show a normal or mildly low level of whole-kidney GFR, which is equal to single-nephron GFR multiplied by nephron numbers^47^. Therefore, results from our study still provides evidence on the relation between higher eGFR and subsequent eGFR changes. Finally, patients with GFR lower than 45 ml/min/1.73 m^2^ or UPCR≧1000 mg/gm were transferred to the Pre-ESRD program for further management. Therefore, informative censoring may be a concern, since the information from those with rapidly deteriorating kidney function were selectively missing. This may be regarded as a problem with the truncated data, since GFR lower than a threshold was unavailable. This is likely to lead to a decrease in the variance of GFR with the increase in follow-up of the cohort, yielding a spurious, negative correlation between the baseline GFR and changes in GFR^25^. Therefore, the negative correlation between GFR at baseline and GFR changes in patients with CKD and DM may be weaker than what has been observed.

## Conclusion

In conclusions, a significantly negative correlation between GFR at baseline and GFR changes was found in patients with CKD and DM, but no such correlation was found in non-DM patients with CKD when correct statistical analyses were undertaken. Higher baseline eGFR or renal hyperfiltration may be only a subclinical indicator but not the major cause of renal damage in patients with DM and early stage CKD. Our findings suggest that higher baseline GFR was associated with a greater GFR decline in DM patients but not in non-DM patients. Investigations about baseline value to subsequent changes should describe model specifications in detail to assure resolving mathematical coupling and then prevent from a spurious conclusion.
